# Acoustic triggered nanobomb for US imaging guided sonodynamic therapy and activating antitumor immunity

**DOI:** 10.1080/10717544.2022.2095058

**Published:** 2022-07-10

**Authors:** Mengmeng Li, Ya Zhu, Chao Yang, Mi Yang, Haitao Ran, Yefeng Zhu, Wei Zhang

**Affiliations:** a Department of Obstetrics and Gynecology, the Second Affiliated Hospital of Chongqing Medical University, Chongqing, China; b Department of Obstetrics and Gynecology, Chongqing Traditional Chinese Medicine Hospital of Jiulongpo District, Chongqing, China; c Department of Radiology, Chongqing General Hospital, Chongqing, China; d Department of Ultrasound, the Second Affiliated Hospital of Chongqing Medical University & Chongqing key Laboratory of Ultrasound Molecular Imaging, Chongqing, China

**Keywords:** Sonodynamic, reactive oxygen species, nanobomb, immunogenic cell death, cancer therapy

## Abstract

We fabricated an ultrasound activated ‘nanobomb’ as a noninvasive and targeted physical therapeutic strategy for sonodynamic therapy and priming cancer immunotherapy. This ‘nanobomb’ was rationally designed via the encapsulation of indocyanine green (ICG) and perfluoropentane (PFP) into cRGD peptide-functionalized nano-liposome. The resulting Lip-ICG-PFP-cRGD nanoparticle linked with cRGD peptide could actively targeted ID8 and TC-1 cells and elicits ROS-mediated apoptosis after triggered by low-intensity focused ultrasound (LIFU). Moreover, the phase change of PFP (from droplets to microbubbles) under LIFU irradiation can produce a large number of microbubbles, which act as intra-tumoral bomber and can detonate explode tumor cells by acoustic cavitation effect. Instant necrosis of tumor cells further induces the release of biologically active damage-associated molecular patterns (DAMPs) to facilitate antitumor immunity. More important, the ‘nanobomb’ in combination with anti-PD-1checkpoint blockade therapy can significantly improve the antitumor efficacy in a subcutaneous model. In addition, the liposomes may also be used as an imaging probe for ultrasound (US) imaging after being irradiated with LIFU. In summary, the US imaging-guided, LIFU activated ROS production and explosion ‘nanobomb’ might significantly improve the antitumor efficacy and overcome drug resistance through combination of SDT and immunotherapy, we believe that this is a promising approach for targeted therapy of solid tumor including ovarian cancer.

## Graphical Abstract

This ‘nanobomb’ was rationally designed via the encapsulation of ICG and PFP into cRGD peptide-functionalized nano-liposome. The US imaging-guided, LIFU activated ROS production and explosion ‘nanobomb’ can significantly improve anti-cancer efficacy and overcome drug resistance through combination of SDT and immunotherapy.

**Figure:**
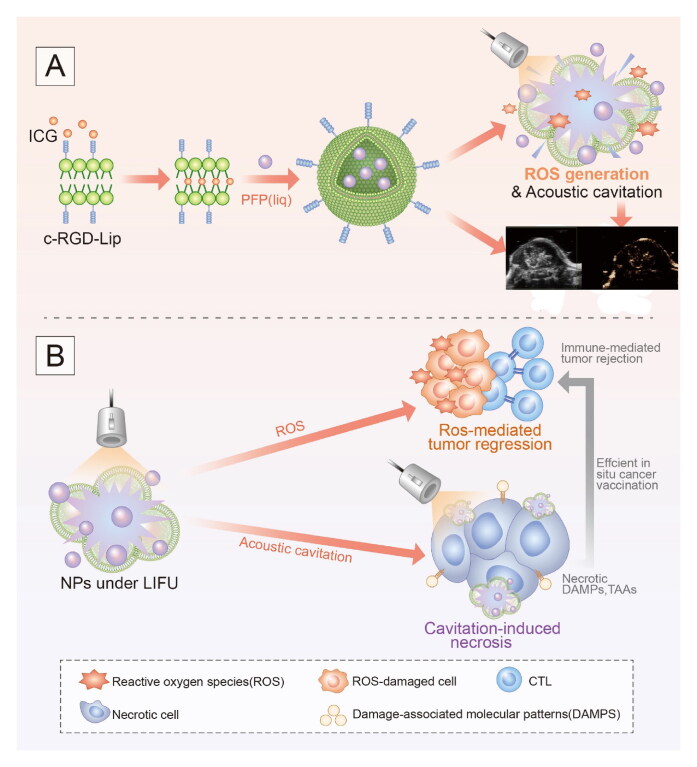

## Introduction

Over the last decade, ovarian cancer has become one of the most lethal gynecological malignancies with high incidence, poor prognosis, mortality rates, and resistance to conventional therapies (Torre et al., 2015; Srivastava et al., 2017; Siegel et al., 2021). According to previous research, up to 30% of ovarian cancer patients die within five years due to relapse and distant metastatic development (Siegel et al., 2021). Consequently, various approaches need to be explored to provide better clinical treatment. Chemotherapy and radiation are two conventional cancer treatments that can reduce cell viability and induce tumor cancer apoptosis by destroying cellular organelles and inhibiting cell division (Erwig & Henson, 2008). In recent years, with the increasing understanding of cancer and its interaction with the immune system, cancer immunotherapy, which trains or stimulates the body’s innate immune system to attack tumor cells, has made rapid progress and shows great promise as the next generation of cancer treatment strategies (Palucka & Banchereau, 2012; Liu et al., 2018). Accumulating evidence suggests that additional engagement of the immune response contributes significantly to the over antitumor efficacy of cancer therapy (Galluzzi et al., 2012; Yoon et al., 2018): for instance, certain chemotherapeutic agents (e.g., oxaliplatin and anthracyclines) and exogenous stimulus (e.g., photodynamic therapy) induce immunogenic cell death (ICD) in tumor cells (Huang et al., 2019; Zhang et al., 2021). Apoptosis has been considered as a procedural form of cell death with poor immunogenicity and physiology (Erwig & Henson, 2008; Tesniere et al., 2008; Green et al., 2009; Morioka et al., 2019). However, Immunogenic cell death is unique in that it can elicit an immune response by exposing or releasing molecules, such as cytokines, chemokines, tumor-associated antigens (TAAs), and damage-associated molecular patterns (DAMPs), including exposure of calreticulin (CRT) at cell surface and release of high mobility group box 1 (HMGB1) and adenosine triphosphate (ATP) (Galluzzi et al., 2017; Montico et al., 2018). Adjuvant immunogenicity provides a new dimension for anti-cancer, and enhanced synergistic therapy is proposed (Huang et al., 2019; Chen et al., 2021). However, despite the promise, current immunotherapies are limited and cannot address all types of tumors. This is probably due to the fact that a single therapeutic strategy is not sufficient to overcome complex antitumor immunity. Therefore, it is necessary to explore safe and efficient comprehensive therapy for effective antitumor therapy.

SDT, which combines low-intensity ultrasound with sonosensitizers, has been investigated as a potential cancer treatment modality (Son et al., 2020; Deng et al., 2021). Sensitizers, such as photosensitizers or sonosensitizers, have recently been shown to destroy cancer cells by generating reactive oxygen species (ROS) in response to light or sound stimulation and by eliciting inflammatory responses during cell death (Sang et al., 2019). Moreover, SDT demonstrates a high propensity for concentrating ultrasound energy to trigger sonosensitizers in far deeper tissue areas and to induce the sonosensitizer’s local cytotoxicity when adequate sonosensitizer accumulation occurs at the tumor site (Chen et al., 2016; Qian et al., 2016). Therefore, SDT has become a promising approach to treat tumors, especially deep-seated tumors. ICG has been identified as a possible sonosensitizer with minimum toxicity and has been approved by the United States Food and Drug Administration (FDA), exhibiting a low incidence of adverse side effects and can be administered directly for diagnostic purposes (Son et al., 2020). Compared to free ICG, supramolecular systems such as nanoparticles have been demonstrated to significantly increase ROS production when exposed to exogenous energy sources (Sheng et al., 2018; Lin et al., 2019).

**Scheme 1.**
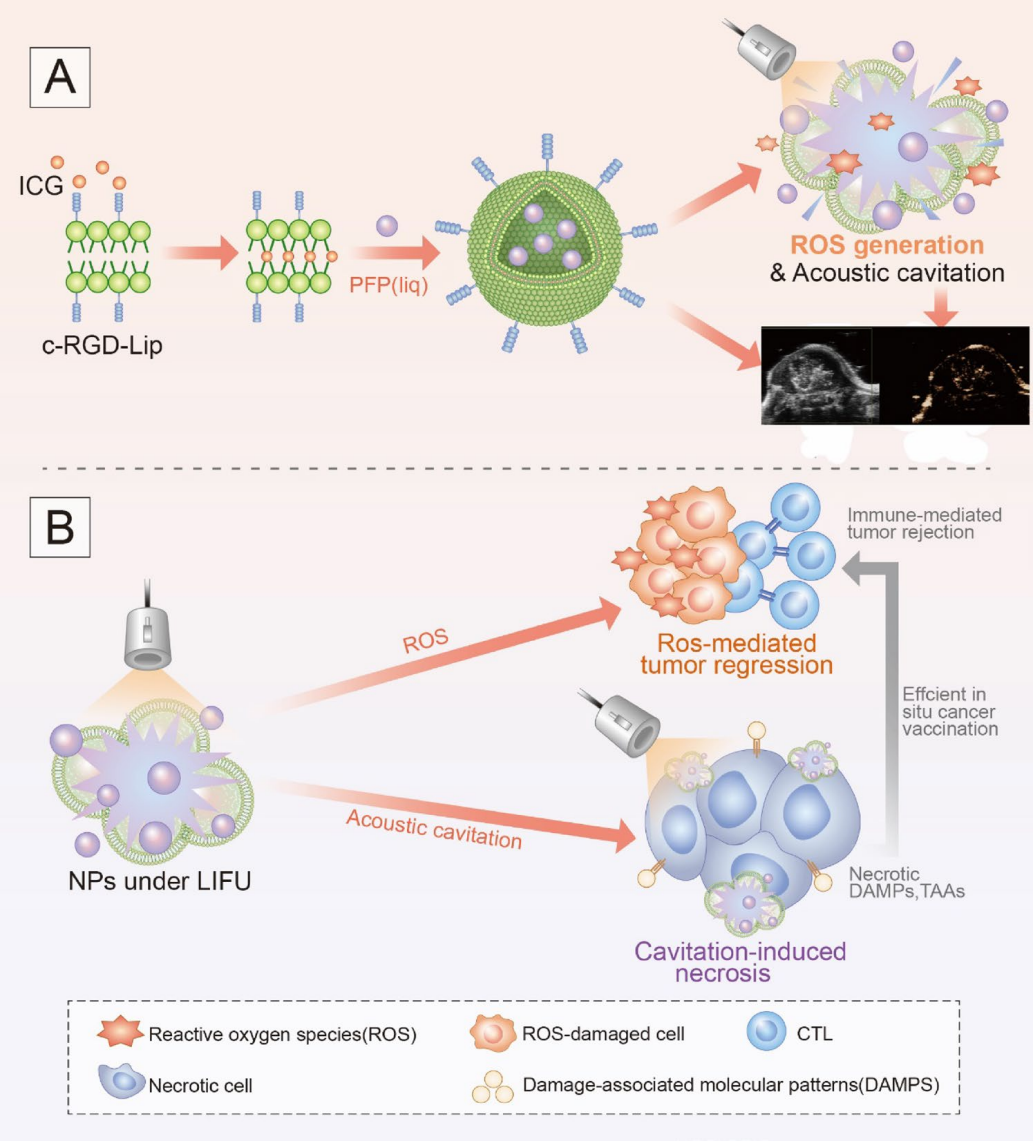
Schematic illustration of acoustic triggered ‘Nanobomb’ for US imaging guided SDT and antitumor immune response.

Given that, we fabricated an ultrasound activated ‘nanobomb’ as a noninvasive and targeted physical therapeutic strategy for sonodynamic therapy and inducing immunogenic cell death. This ‘nanobomb’ was rationally designed *via* the encapsulation of indocyanine green and perfluoropentane into cRGD peptide-functionalized nano-liposome. The resulting Lip-ICG-PFP-cRGD nanoparticle linked with cRGD could actively targeted ID8 and TC-1 cells and elicits ROS-mediated apoptosis after triggered by ultrasound. Moreover, the phase change of PFP (from droplets to microbubbles) under low-intensity focused ultrasound irradiation can produce a large number of microbubbles, which act as intra-tumoral bomber and can detonate explode tumor cells by acoustic cavitation effect (Kiessling et al., 2014; Zhang et al., 2015), resulting in the release of biologically active DAMPs (CRT and HMGB1) to facilitate antitumor immunity (Scheme 1). In addition, our liposomes may also be used as an imaging probe for US imaging after being irradiated with LIFU. Such nanoparticles provided us with therapeutic and diagnostic vehicles to destroy cancer cells and improve antitumor efficacy when combined with SDT and immunotherapy.

## Materials and methods

### Materials

Liposome includes 1,2-dipalmitoyl-sn-glycero-3-phosphocholine (DPPC), 1,2-distearoyl-sn-glycero-3-phosphoethanolamine-N-[methoxy(polyethyleneglycol)-2000] (DSPE-PEG2000), 1, 2-distearoyl-sn-glycero-3-phospho-(1′-rac-glycerol) (DSPG), and cholesterol were purchased from Avanti Polar Lipids (Alabaster, AL, USA). Perfluoropentane (PFP), indocyanine green (ICG), and propidium iodide (PI) were obtained from Sigma Aldrich (St. Louis, MO, USA).

Reactive Oxygen Species Assay Kit (20,70-dichlorofluorescin diacetate, DCFH-DA) and 1,10-dioctadecyl-3,3,30,30-tetramethylindocarbocyanine perchlorate (DiI) were purchased from Santa Cruz Biotechnology (TX, USA). All reagents were of analytical grade and used without further purification. ID8 cells and cervical-cancer-related TC-1 cells were purchased from BeNa Culture Collection (Beijing, China).

### Preparation of Lip-ICG-PFP-cRGD, Lip-ICG-PFP

The liposomes loaded with ICG and PFP, referred to as Lip-ICG-PFP-cRGD, were synthesized by a two-step emulsion method as described previously. Briefly, 5.0 mg of DPPC, 2.0 mg of DSPG, 1.5 mg of DSPE-PEG-cRGD, and 1.5 mg of cholesterol were dissolved in 10 mL of methanol and 10 mL of chloroform. The solution was then transferred to a round bottom flask to form lipid films by rotary evaporation. After that, 2 mL of phosphate buffer saline (PBS) was added to the flask, sonicating the mixture to hydrate the lipid films for 5 min using ultrasound cleaner. Next, 0.05 mL of ICG aqueous solution (10 mg/mL) and 0.2 mL of PFP were added into lipid films and emulsified in an ice bath for 3 min using a sonicator (Heat System Inc, USA). Following that, a solution of Lip-ICG-PFP-cRGD was purified three times by centrifugation (8000 rpm, 5 min), and the supernatant was removed. Finally, 2 mL of PBS was added to the precipitate for further experimentation. The Lip-ICG-PFP liposomes were synthesized by sonicating the hybrid of lipid films without cRGD in an ice bath for 3 min and then purified three times by centrifugation (8000 rpm, 5 min).

### Characterization of liposome

Nanoparticles (NPs) carrying PFP and ICG were successfully synthesized using DSPG, DSPE-PEG2000, DPPC, and cholesterol, and were called Lip-ICG-PFP-cRGD, generating a US-mediated cavitation effect. The diameter, zeta potential, and polydispersity index (PDI) of different kinds of NPs were measured by Zetasizer Nano ZS unit (Malvern Instruments, Malvern, UK) and their morphology and structure were observed by transmission electron microscopy (TEM). Ultraviolet–visible (UV–vis) spectrophotometer with scanning wavelength ranging from 200 to 900 nm was used to determine the absorption spectra of different nanoparticles. UV–vis spectrophotometer (260-Bio, Thermo Fisher Scientific) was used to evaluate the entrapment efficiency and loading of ICG, and entrapment efficiency and loading content were determined based on Eqs. (1) and (2):

(1) ICG loading (%) = ICG amount in NPs/mass of LIPC NPs × 100%

(2) Entrapment efficiency (%) = ICG amount in LIPC NPs/ICG feeding × 100%

### LIPC NPs: Lip-ICG-PFP-cRGD nanoparticles

#### Detection of ROS

1, 3-Diphenylisobenzofuran (DPBF) was used to determine ROS generation in nanoparticles. A total of 60 µL of DPBF (4 mg/mL), 50 µL of Lip-ICG-PFP-cRGD, and 1 mL of double distilled water were added into Eppendorf tube and exposed to low-intensity ultrasound with 2 W/cm^2^ for 0–10 min in dark. Then, a multimode reader was used to evaluate ROS production by measuring the absorption at 410 nm.

To detect intracellular ROS generation, 2′, 7′-dichlorofluorescin diacetate (DCFH-DA) was employed as an indicator. In brief, 1 × 10^5^ ID8 cells were seeded in 12-well plates and co-incubated with Lip-ICG-PFP-cRGD (the concentration of each group was 0.4 mg/mL) for 3 h. All the wells were divided into six groups: control group, NPs group, only LIFU group (2 W/cm^2^ for 60 sec), and NPs combined with LIFU group (2 W/cm^2^ for 60 s). Then, each of the wells was washed three times with PBS. Subsequently, the cells were cultured with diluted DCFH-DA for 30 min in dark. The ROS generation was evaluated by flow cytometry.

ID8 cells were seeded in confocal dishes at a density of 1 × 10^5^ for 24 h, and the rest of routine was as described above. Finally, laser scanning confocal microscopy (LSCM, Nikon AJR, Japan) was used to detect ROS generation.

### Determination of cellular uptake

ID8 cells and TC-1 cells were seeded onto confocal dishes at a density of 1 × 10^5^ for 24 h. Next, the original media was replaced by media containing Lip-ICG-PFP or Lip-ICG-PFP-cRGD labeled with DiI dye for co-incubation with different periods (1.0, 2.0, and 3.0 h). The cellular uptake was evaluated by flow cytometry or confocal laser scanning microscopy. The cells were washed with PBS and cultured with 2-(4-amidinophenyl)-6-indolecarbamidine dihydrochloride (DAPI) for 10 min to stain the cell nuclei. Subsequently, PBS was used to wash the cells three times, and 4% paraformaldehyde was utilized to fix the cells. Finally, the cellular uptake ability was evaluated by confocal laser scanning microscopy.

### US imaging *in vitro* and *in vivo*

The agar-gel model (3% agar w/v in double-distilled water) was utilized to evaluate Lip-ICG-PFP-cRGD capacity, which acts as contrast agents for ultrasound imaging. For US imaging, liposomes were exposed to low-intensity focused ultrasound (2–6 W) for 100–300 sec. The contrast-enhanced ultrasound (CEUS) and B-mode images were obtained by an ultrasonic diagnostic instrument (MyLab 90; Esaote, Italy).

All of the animal experiments were conducted with the approval of the Institutional Animal Care and Use Committee of the Animal Experiment Center of Chongqing Medical University. TC-1 cancer baring mice were intravenously injected with Lip-ICG-PFP (200 µL, 1 mg/mL) or Lip-ICG-PFP-cRGD (200 µL, 1 mg/mL), then the B-mode and CEUS images were observed using ultrasonic diagnostic instrument at 24 h after injection.

### Cell viability and cell death assay using annexin V/PI staining

To assess cytotoxicity *in vitro*, ID8 and TC-1 cells were seeded in a 96-well plate for 24 h, and then, 100 µL of Lip-ICG-PFP-cRGD nanoparticles with diverse concentrations were added and cultured for another 24 h. Cell viabilities were tested by the CCK-8 assay.

To evaluate the therapeutic effects, ID8 and TC-1 cells were seeded in a 96-well plate and co-cultured with Lip-ICG-PFP-cRGD nanoparticles (different concentrations were divided into five groups) for 3 h. PBS was utilized to wash the wells three times, and the medium in each well were displaced with fresh medium. Next, the groups were exposed to low-intensity ultrasound with various power (2.0 W/cm^2^) for 200 sec in the dark, and cell viabilities were determined using the CCK-8 method.

ID8 cells were cultured in 6-well dishes for 24 h. Additionally, cells were incubated with liposomes or free ICG for 3 h and washed with PBS. Then, cells were trypsinized and re-suspended with 400 µL of 1% FBS containing annexin V binding buffer. After that, the cells were exposed to LIFU for 200 sec (power 2.0 W/cm^2^). The cell viabilities were further incubated for 1 h and stained with FITC annexin V and PI for 10 min. Finally, the cells were observed using a fluorescence microscope and analyzed with flow cytometry.

### The CRT expression and HMGB1 release assay

The ID8 cells were co-cultured with different nanoparticles (Lip-ICG-cRGD or Lip-ICG-PFP-cRGD, 0.8 mg/mL) in 6-well plates for 3 h. All the wells were divided into four groups: control group, only LIFU group, only Lip-ICG-PFP-cRGD group, Lip-ICG-cRGD + LIFU group, and Lip-ICG-PFP-cRGD + LIFU group (2 W/cm^2^ for 200 sec). The cells were cultured for another 18 h, and the supernatant was added into another 6-well plate which was cultured with dendritic cells (DC) for 6 h. Subsequently, the cells and supernatants from DC co-cultured were collected to examine the CRT and HMGB1 level by ELISA (LifeSpan BioSciences), according to manufacturer’s instructions.

### The expression of CD86 in dendritic cells

To investigate the expression of CD86 (a marker for DC maturation), ID8 cells were seeded and co-incubated with different nanoparticles. Following exposure to different treatments, the supernatant was collected separately and added into another 12-well plate cultured with DC for 24 h. After washing with PBS and staining with FITC-conjugated anti-CD86 for 60 min in the dark, the samples were analyzed using flow cytometry to quantify CD86 expression in DC.

### Antitumor effect *in vivo*

When the tumor volume reached about 80 mm^3^, TC-1 cancer bearing C57 mice (6-8 weeks) were randomly divided into 5 groups (*n* = 5): i) PBS, ii) LIFU, iii) Lip-ICG-PFP-cRGD, iv) Lip-ICG-PFP-cRGD + LIFU, and v) Lip-ICG-PFP-cRGD + LIFU + anti-PD-1. 200 µL of Lip-ICG-PFP-cRGD (1 mg/mL) were administered on day 1 and 3, respectively. In addition, LIFU (2 W, 600 sec, duty cycle 40%) was applied to the tumors after at 24 h post injection, and anti-PD-1 antibody (100 µg per mice) was intraperitoneally injected after an additional day. Tumor volume and mice weight were measured every two days.

### Statistical analysis

The experimental data were analyzed using GraphPad Prism (version 8.0) with the unpaired Student’s *t*-test, or the paired Student’s *t*-test. *P*-values < .05 indicated statistically significant difference (*p < .05, **p < .01).

## Results and discussion

### Characterization

Previous study developed a robust stealthy phospholipid liposome that demonstrated long circulation and could be stimulated by NIR light to release encapsulated drugs (Ge et al., 2019). Compared to NIR irradiation, US achieves much deeper penetration, and its energy can be regulated in exposed areas.

**Figure 1.**
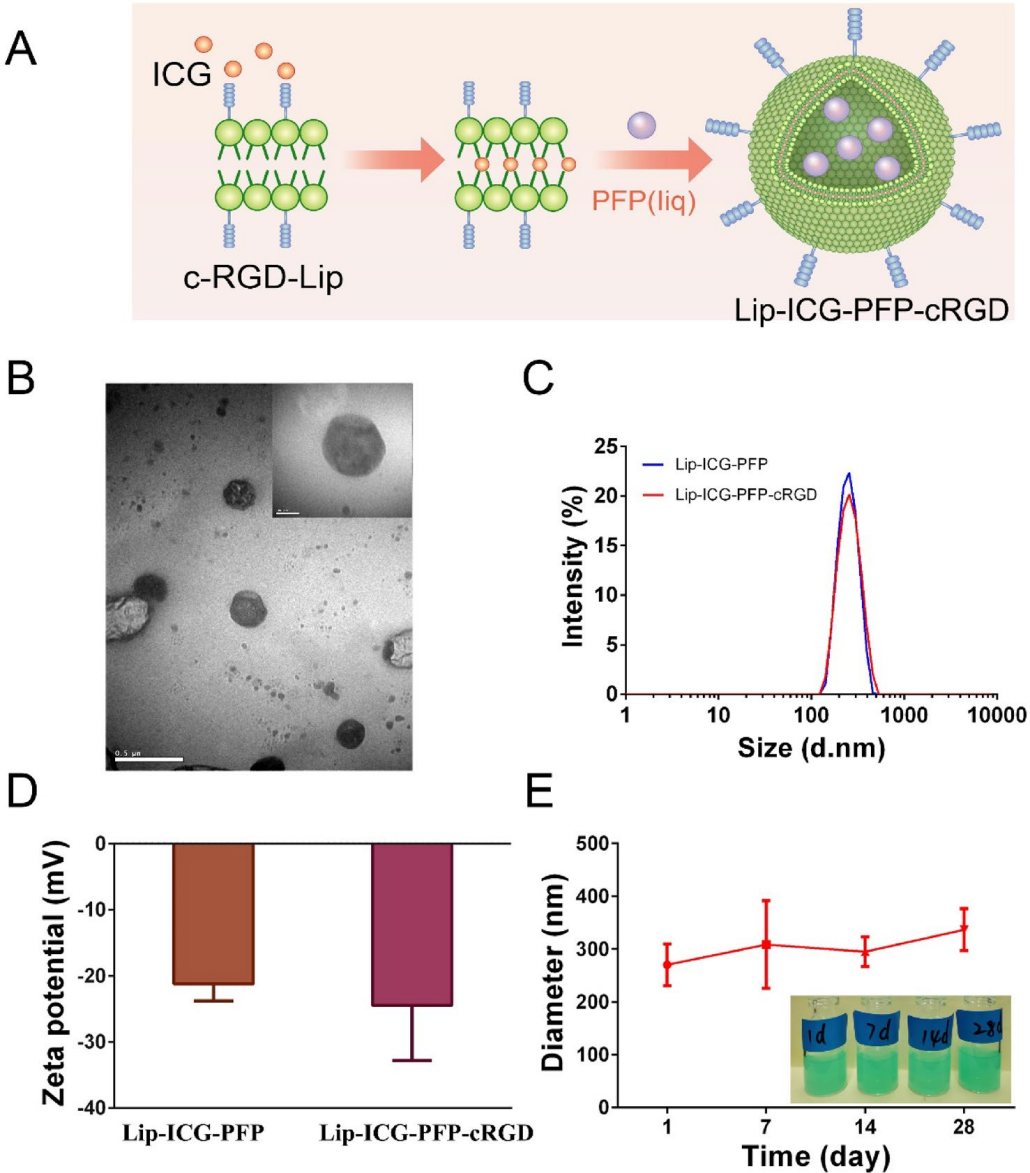
Characterization of nanoparticles. (A) Schematic diagram for the fabrication of Lip-ICG-PFP-cRGD. (B) TEM images of Lip-ICG-PFP-cRGD. (C) Size distribution of Lip-ICG-PFP and Lip-ICG-PFP-cRGD. (D) Zeta potential of the Lip-ICG-PFP and Lip-ICG-PFP-cRGD. (E) Size changes of Lip-ICG-PFP-cRGD at 4 °C for 28 days (*n* = 3).

As shown in Figure 1(A), the liposomes were synthesized using a two-step emulsion method (Zhao et al., 2020). Transmission electron microscopy demonstrated a nearly spherical structure of Lip-ICG-PFP-cRGD with smooth surfaces (Figure 1(B)). Dynamic light scattering measurements manifested a mean diameter of ∼300 nm (PDI 0.135) with PFP incorporation, indicating uniform particle size distributions (Figure 1(C)). Zeta potentials of Lip-ICG-PFP and Lip-ICG-PFP-cRGD were −21.22 ± 2.54 mV and −24.49 ± 8.31 mV (Figure 1(D)) at pH = 7.4, respectively. Furthermore, Lip-ICG-PFP-cRGD could maintain appreciable stability in cell culture medium at 4 °C for 28 days (Figure 1(E)) without significant diameter changes, suggesting excellent long-term stability.

**Figure 2.**
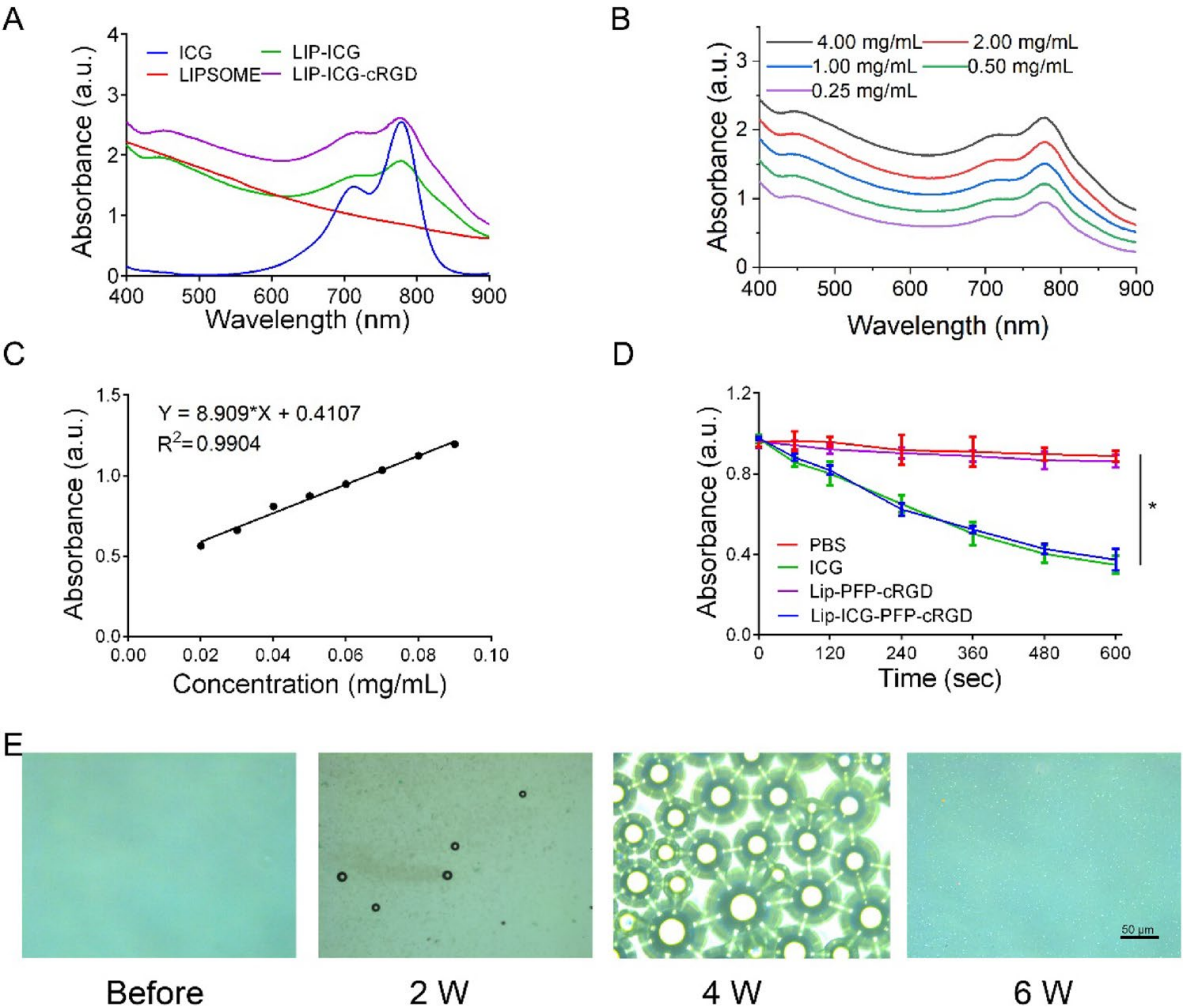
(A) Identification of liposomes by UV–vis spectroscopy. (B) UV–vis spectra of Lip-ICG-PFP-cRGD in different concentrations. (C) The concentration-absorbance standard curve of ICG. (D) DPBF consumption of different nanoparticles under LIFU irradiation (*n* = 3, *p < .05). (E) Optical images of Lip-ICG-PFP-cRGD after LIFU irradiation (2 min, 2–6 W).

The UV–vis absorption spectra (Figure 2(A)) of Lip-ICG, Lip-ICG-PFP and Lip-ICG-PFP-cRGD displayed prominent absorption peaks of ICG at 790 nm, indicating that ICG was successful encapsulated into nanoparticles. A similar result was obtained that the absorbance of Lip-ICG-PFP-cRGD increased with concentration (Figure 2(B)). To calculate the encapsulation efficiency of ICG in liposomes, a standard curve was drawn (Figure 2(C)), and ICG amount encapsulated in Lip-ICG-PFP-cRGD was determined spectrophotometrically as a 92% loading efficiency. The individual structure of Lip-ICG-PFP-cRGD was discovered to encapsulate vaporable PFP, which generated nanobubbles and fused into microbubbles after triggering by LIFU. As ICG is one of the promising forms of sonosensitizers, 1, 3-Diphenylisobenzofuran (DPBF) was used to test ROS production. Since the absorbance intensity of DPBF is reduced at 410 nm in UV–vis spectrum after oxidation by ROS, DPBF consumption assists in calculating ROS production. As displayed in Figure 2(D), DPBF consumption in free ICG group and Lip-ICG-PFP-cRGD plus LIFU group was notably higher compared to that in PBS or Lip-PFP-cRGD group, and with prolonged irradiation time, the Lip-ICG-PFP-cRGD group showed obviously higher ROS production efficacy, suggesting its potential as sonosensitizer for SDT.

The PFP encapsulated inside the liposome was converted into numerous microbubbles within 2 min when treated with LIFU and was visualized using optical microscopy (Figure 2(E) and S1). As the power increased, nanoparticles with PFP cores could be converted into microbubbles. The adjacent microbubbles could fuse with each other to form larger microbubbles until they burst, consistent with previous studies, revealing the excellent ability to function as an US imaging agent and acoustic cavitation eliciting necrosis.

### Targeting efficiency *in vitro*

**Figure 3.**
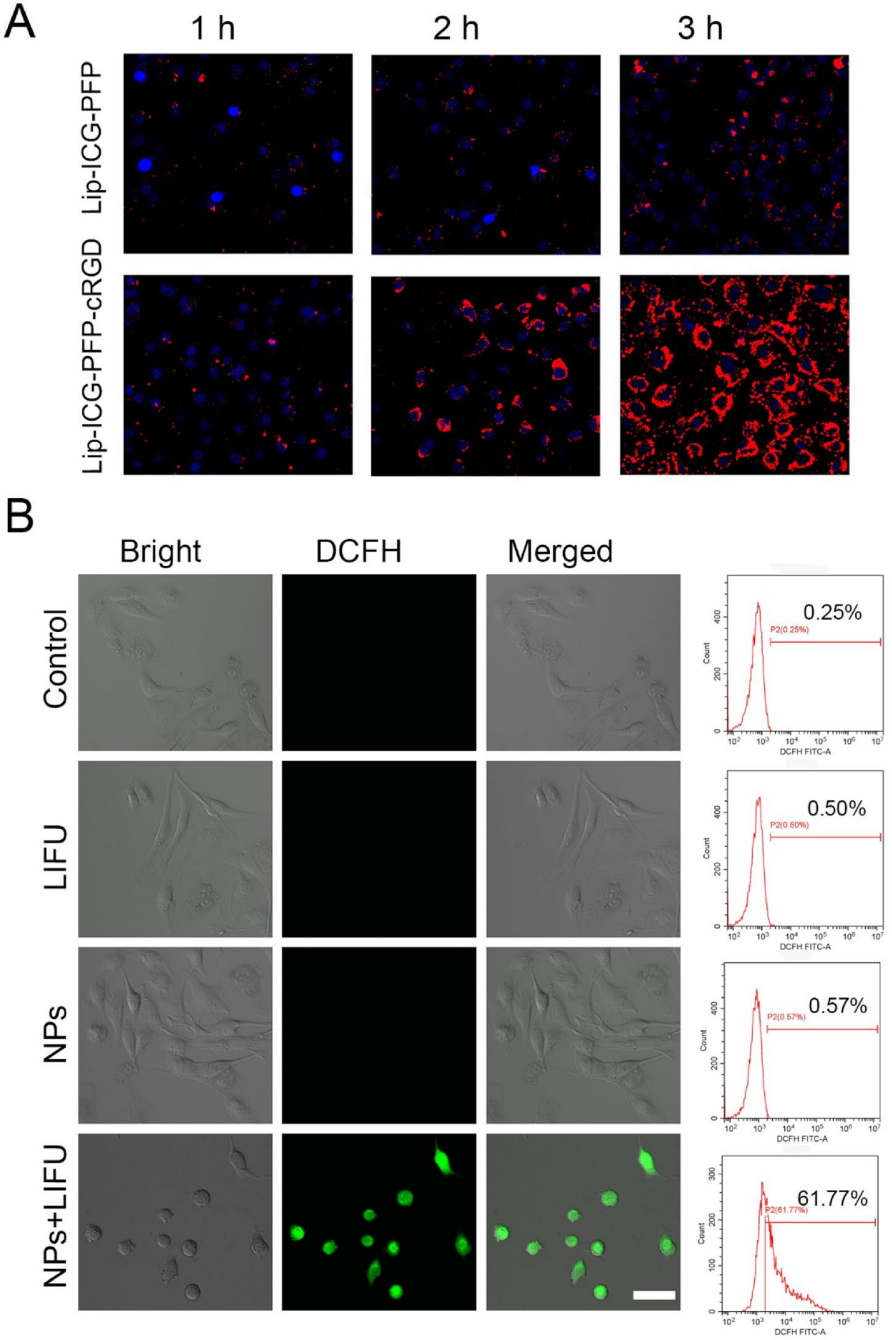
(A) CLSM images of ID8 cells after incubation with Lip-ICG-PFP-cRGD and Lip-ICG-PFP nanoparticles for elevated time. (B) CLSM images and flow cytometry analysis of ROS generation in ID8 cells treatment with various treatments, as detected with DCFH-DA (NPs: Lip-ICG-PFP-cRGD, Scale bar:50 µm).

Excellent antitumor effects require effective cellular uptake of medicine by cancer cells. Therefore, we evaluated the uptake capacity of prepared nanoparticles by ID8 and TC-1 cells. The efficacy of DiI-labeled liposomes to target tumor cells was verified by CLSM and flow cytometry. As presented in Figure 3(A) and S2, the fluorescence intensity was almost equally weak in cancer cells after 1 h of incubation with Lip-ICG-PFP and Lip-ICG-PFP-cRGD, and a higher level of cellular uptake could be observed when the incubation time increased from 2 to 3 h. Considerable fluorescence intensity in cells incubated with Lip-ICG-PFP-cRGD for 3 h was observed, whereas a much weaker fluorescent signal was exhibited in cells incubated with Lip-ICG-PFP. This result indicating that cRGD can lead to a higher cell internalization of drugs.

### ROS generation in cells

To detect the intracellular mechanism of Lip-ICG-PFP-cRGD as sonosensitizers in destroying cancer cells, intracellular ROS levels were tested using DCFH-DA, which could be converted from non-fluorescence status into fluorescent 2,7-dichlorofluorescein (DCF) by ROS. It can be found that Lip-ICG-PFP-cRGD combined with LIFU irradiation induced a large amount of intracellular ROS production, which demonstrated strong green fluorescence in ID8 cells (Figure 3(B)). In contrast, neither control cells nor US irradiation induced the intracellular fluorescence. Moreover, similar results were also verified again by the quantitative analysis with flow cytometry, indicating that Lip-ICG-PFP-cRGD could efficiently generate ROS for effective SDT. It can be deduced that nanoparticles as sonosensitizers can generate ROS under LIFU irradiation to induce the toxic effect and achieve the therapeutic function afterward.

### *In vitro and in vivo* ultrasound imaging

**Figure 4.**
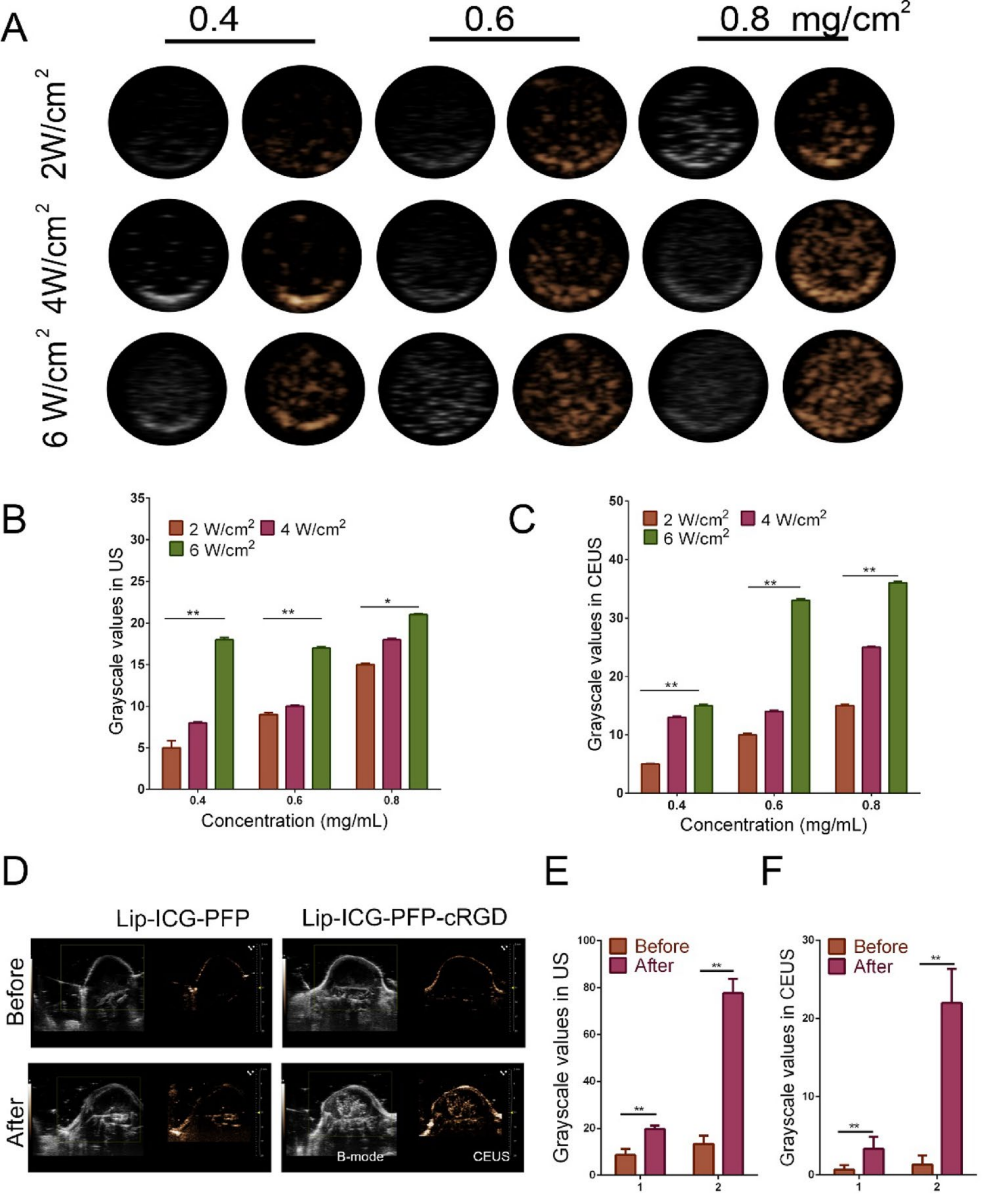
(A) B-mode and CEUS images of Lip-ICG-PFP-cRGD after LIFU irradiation under different conditions (2-6 W/cm^2^, 1 min). (B, C) The grayscale value in B-mode and CEUS after irradiated with LIFU (the data are shown as mean ± SD, *n* = 3). (D) Ultrasound imaging of tumors in B-mode and CEUS mode before and after LIFU irradiation. (E, F) The grayscale value of tumor in B-mode and CEUS mode before and after irradiated with LIFU in different treatments (1: Lip-ICG-PFP, 2: Lip-ICG-PFP-cRGD).

The individual structure of liposome was creatively discovered to encapsulate vaporable PFP, which generated nanobubbles and fused into microbubbles after triggering by LIFU, thus realizing the enhanced US imaging. Thus, it was interesting to investigate the echogenic property of the nanoparticles with US irradiation *in vitro*. We speculated that phase-changeable liposomes could serve as ultrasound agents to enhance US imaging. US images of liposome demonstrated acoustic reflectivity contrast contributing to the generation of gas bubbling under US irradiation, accord with quantitative production results (Figure 4(A–C)). In this regard, enhanced US imaging showed that Lip-ICG-PFP-cRGD was considered an US contrast agent.

Based on the good ultrasound imaging performance of Lip-ICG-PFP-cRGD *in vitro*, we further verified the ultrasonic imaging ability *in vivo*. Regular low or anechoic and no contrast enhanced signals were observed in both targeted (Lip-ICG-PFP-cRGD) and non-targeted (Lip-ICG-PFP) group. LIFU were performed 24 h after injection in each group. Compared with the previous image, the targeted group showed significantly stronger speck-like echo signal in both B-mode and CEUS mode, while the non-targeted group showed only weak signal (Figure 4(D)). This suggested that cRGD promotes the accumulation of nanoparticles in tumor tissue, and when acoustic droplet vaporization was performed in the tumor site triggered by LIFU, a large number of microbubbles are generated, thus enhancing ultrasound imaging. DFY software was used to analyze the average gray value in two modes. As shown in Figure 4(E,F), the mean gray scale values in targeted group were significantly higher than that in the non-targeted group, which was consistent with the ultrasound imaging results. These results indicated that targeted nanoparticles were suitable as ultrasound imaging agents and effective nanocarriers *in vivo*.

### Antitumor effect *in vitro* and ICD-induced immune priming

**Figure 5.**
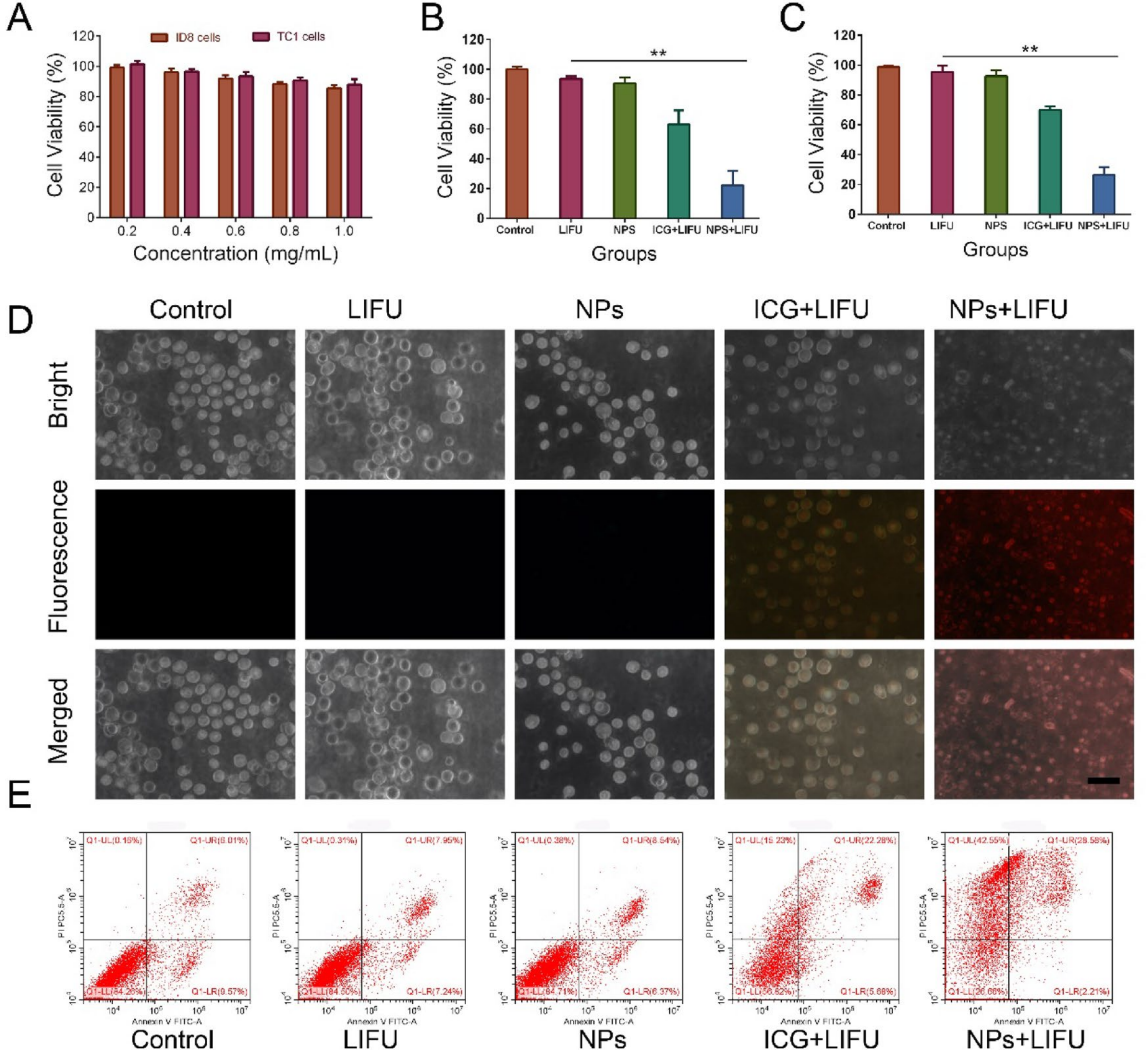
(A) Relative cell viability of ID8 and TC-1 cells after incubation with different concentrations (0.2, 0.4, 0.6, 0.8, and 1.0 mg/mL) of Lip-ICG-PFP-cRGD (NPs). (B) Relative cell viability of ID8 (B) and TC-1 (C) cells after various treatments. (The error bars represent standard deviation, *n* = 3). (D) Fluorescence microscope image of ID8 cells stained annexin V/PI after different treatments (Scale bar =50 µm). (E) Flow cytometry analysis of apoptosis of ID8 cells stained with annexin-FITC and PI after various treatments.

The toxicity of Lip-ICG-PFH-cRGD was measured by CCK-8 assay after co-incubation with ID8 and TC-1 cells for 24 h at different concentrations. As Figure 5(A) displays, after 24 h of incubation, Lip-ICG-PFP-cRGD revealed no significant cytotoxicity on ID8 and TC-1 cells at a concentration up to 1 mg/mL, indicating good cytocompatibility *in vitro*. No apparent cytotoxicity occurs within the therapeutic concentration range employed in this study. Next, to observe synergistic effectiveness of liposomes when irradiated with LIFU. Above all, no obvious cytotoxicities were observed in only LIFU group, indicating that the LIFU irradiation was harmless to ID8 and TC-1 cells. As expected, Lip-ICG-PFP-cRGD + LIFU group displayed the highest cells cytotoxicity against ID8 and TC-1 cells, and more than 60% of cells were killed by cytotoxic ROS and acoustic cavitation, eliciting necrosis in the presence of LIFU (Figure 5(B,C)).

However, the CCK8 method could not completely determine which one should be mainly responsible for the cell death, since besides bubbling-induced instant necrosis, the production of ROS could also cause cell apoptosis. Necrosis differs from apoptosis in morphological features such as cytoplasmic swelling and cell membrane rupture. However, cell shrinkage, nuclear fragmentation, and formation of apoptotic bodies are characteristic manifestations of apoptosis (Vanden Berghe et al., 2010). The annexin V/propidium iodide (PI) assay was implemented by treating the samples with ID8 cancer cells (Figure 5(D,E)) to appraise the cell death mechanism. When the cells were treated with liposome or LIFU only, no obvious fluorescent signals from annexin V or PI were observed, indicating that there was no apparent morphology change of membrane, further suggesting that LIFU irradiation and nanoparticles have no significant damage to the cells. However, apoptotic characteristics (i.e., chromatin condensation of cells and externalization of phosphatidylserine) were clearly demonstrated in free ICG plus LIFU treated cells. Interestingly, cells treated with Lip-ICG-PFP-cRGD plus LIFU showed rupture of cell membrane, leakage of cytoplasmic components, membrane debris, and other cell morphological damage features, indicating that Lip-ICG-PFP-cRGD nanoparticles act like bombs, physically damaging cell membranes when irradiated by LIFU. Nanoparticles induced cell membrane disintegration under LIFU irradiation, resulting in membrane fragments, cytosolic components, and leakage of chromatin (Gong et al., 2019). It is worth noting that, the cells showed morphological characteristics of necrosis when they were treated with Lip-ICG-PFP-cRGD plus LIFU irradiation, suggesting that the occurrence of cell necrosis. These results suggest that, Lip-ICG-PFP-cRGD could induce cell necrosis by physical disruption of the cell membrane under LIFU irradiation. This is probably due to the LIFU-triggered, rapid phase transitions of PFP from droplets to microbubbles.

**Figure 6.**
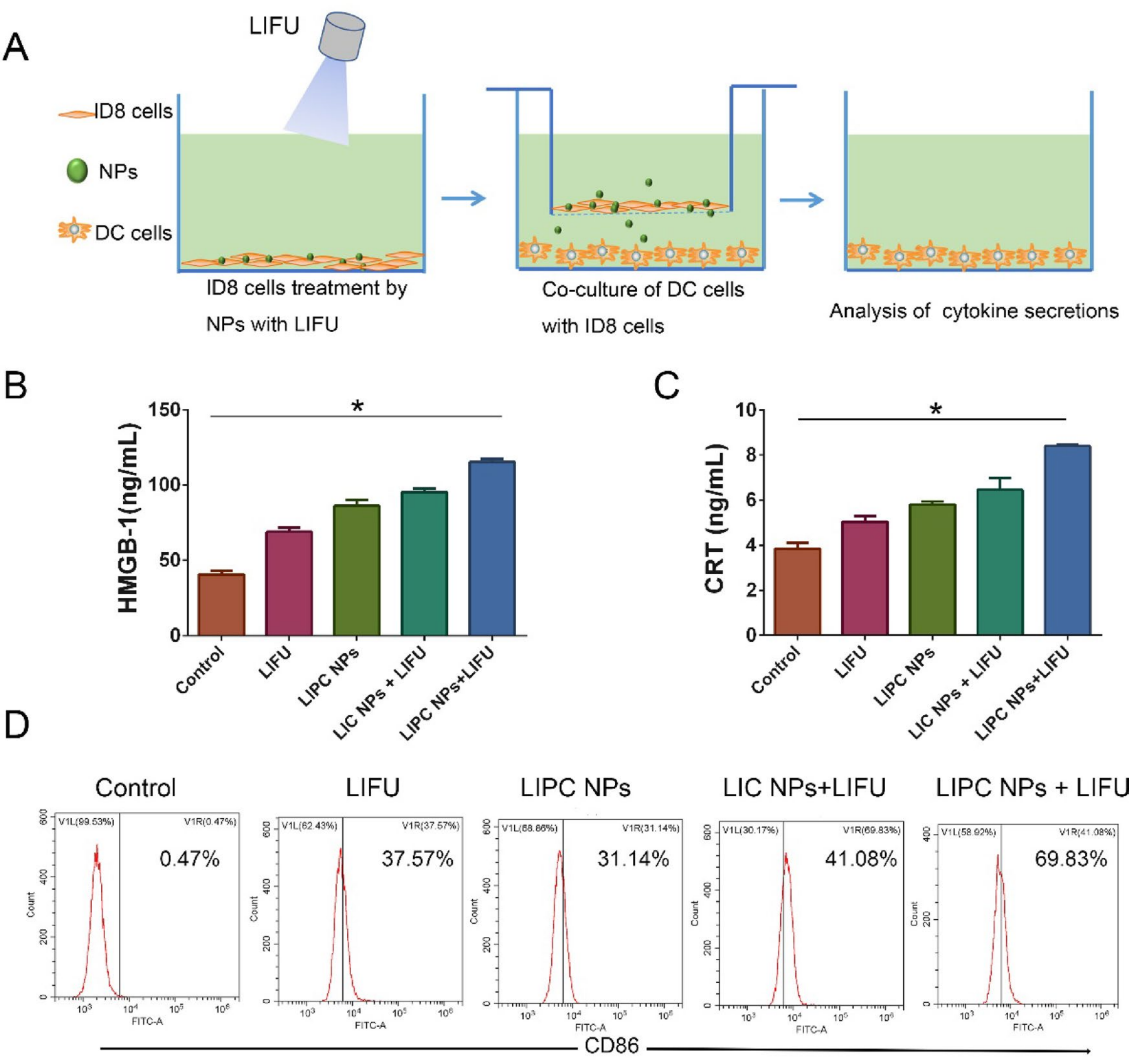
(A) Schematic illustration of the experiments design for the effect on immune responses of LIFU activated ‘nanobomb’. (B, C) Quantitative assay of HMGB1 release and CRT exposure of ID8 cells into the medium using ELISA, ID8 cells were incubated with PBS, Lip-ICG-cRGD (LIC NPs) and Lip-ICG-PFP-cRGD (LIPC NPs) for 3 h and treated with LIFU (2 W/cm^2^) for 200 sec. (The error bars represent standard deviation, *n* = 3; *p < .05). (D) The amount of CD86^+^ DCs through flow cytometry after various treatments *in vitro*.

Unlike apoptosis, necrosis stimulates the release of intact DAMPs from the cells, thus effectively mediating the maturation and activation of dendritic cells (DCs). Of the DAMPs released, HMGB1 has often been considered a necrosis representative marker (Kaczmarek et al., 2013). The effect on immune responses of LIFU activated ‘nanobomb’ was evaluated (Figure 6(A)). As depicted in Figure 6(B), Lip-ICG-PFP-cRGD plus LIFU could induce a significant extracellular release of HMGB1. Additionally, immunogenic cell death (ICD) of tumor cells is characterized by eliciting cell surface expression of pro-apoptotic calreticulin (CRT) (Galluzzi et al., 2017). The exposure of CRT on cancer cell surface serves as an ‘eat me’ signal to antigen-presenting cells (e.g., DCs and macrophages) and trigger an anti-tumor immune response. After treatment of Lip-ICG-PFP-cRGD plus LIFU, CRT exposure on ID8 tumor cells was significantly elevated (Figure 6(C)). To further evaluate the efficacy of turning tumor cells into antigen-presenting cells, DC maturation was investigated. As a result, CD86 expression, a DC maturation marker, remarkably increased via Lip-ICG-PFP-cRGD treatment upon LIFU irradiation compared with other control groups (Figure 6(D)). In conclusion, it was evident that under LIFU irradiation, PFP in Lip-ICG-PFP-cRGD nanoparticles phase transitions from droplets to microbubbles promote cell necrosis, release DAMPs, and ultimately enhance immunogenic cell death.

### Antitumor effect *in vivo*

**Figure 7.**
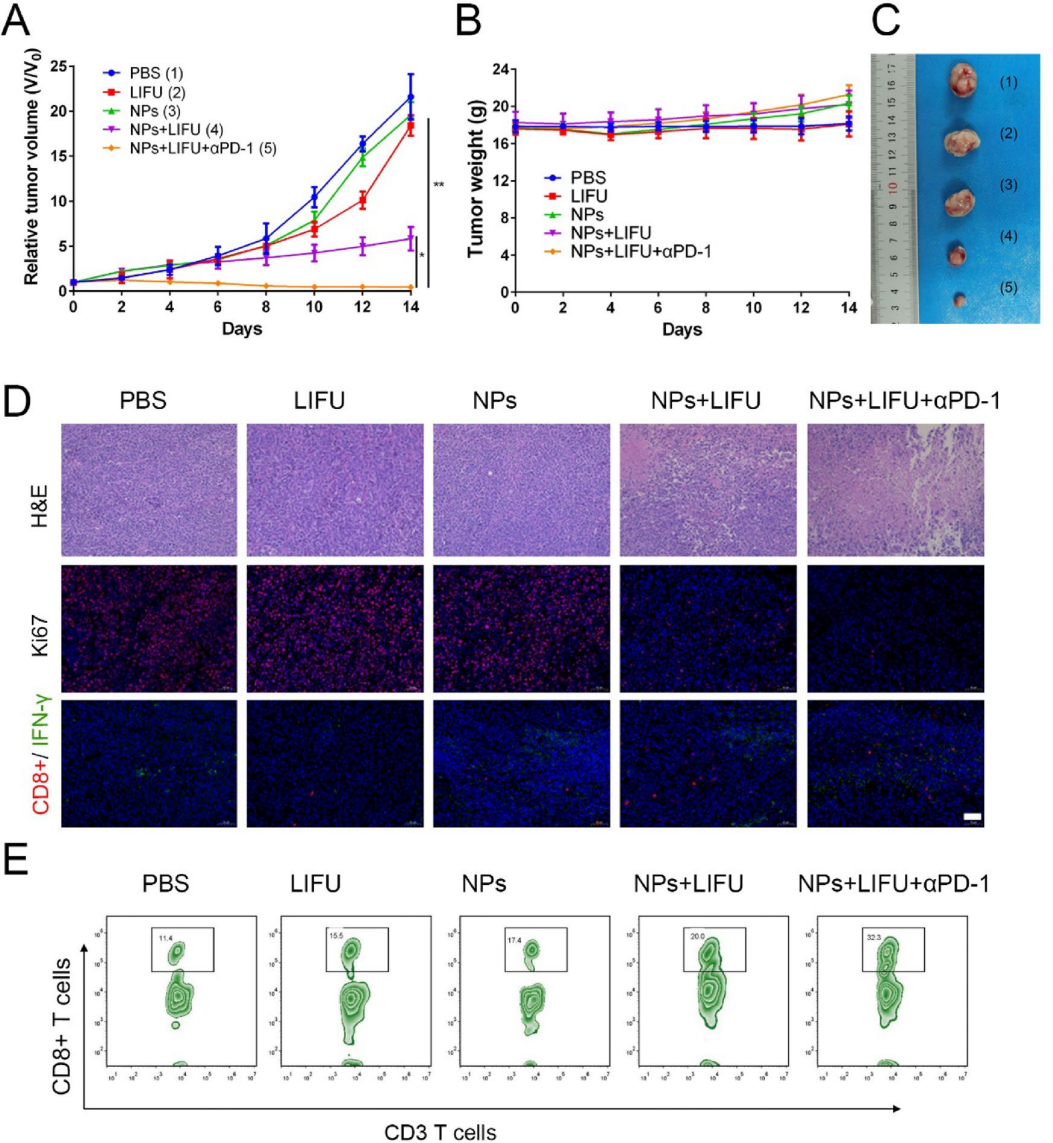
(A) Tumor growth curve of TC-1 tumor-bearing mice with various treatments (*n* = 5). (B) The weight of mice with various treatments (*n* = 5). (C) Representative images of the tumor from each group after euthanizing the animal on day 14. (D) The H&E staining and Ki67, effector CD8^+^T cells, and IFN-γ immunofluorescence images of tumors after different treatment (scale bar: 50 µm). (E) Flow cytometry data of cytotoxic T lymphocytes infiltration in tumors. CD8^+^ T cells were defined as cytotoxic T lymphocytes. (NPs: Lip-ICG-PFP-cRGD nanoparticles).

Encouraged by the ability of ‘nanobomb’ in combination with ultrasound induced necrosis to release bioactive DAMPs and promote the maturation of dendritic cells, we next evaluated its potential to enhance immune checkpoint blockade response by using a TC-1 tumor xenograft in C57 mice model. As shown in Figure 7(A,C), the maximum tumor volume was found in control group, and there was no significant difference in tumor volume in the LIFU group and Lip-ICG-PFP-cRGD alone group compared with the PBS-treated control group, indicating LIFU and Lip-ICG-PFP-cRGD alone had no obvious effect on the tumor growth. However, LIFU combined with ‘nanobomb’ group showed effective tumor inhibition, the enhanced therapeutic efficacy should be due to ROS mediated apoptosis and bubble-induced necrosis. To evaluate whether nanoparticles have the ability to enhance anti-tumor immune response, the anti-tumor effect of ‘nanobomb’ combined with anti-PD-1 antibody was also tested. Notably, Lip-ICG-PFP-cRGD + LIFU + anti-PD-1 group had stronger inhibition of tumor growth without resulting in body weight loss. Lip-ICG-PFP-cRGD combined LIFU and anti-PD-1 significantly enhanced the anti-tumor response, with tumor inhibition rate of about 90%, suggesting PD-1 blockade significantly enhanced ‘nanobomb’-mediated tumor immunotherapy. Furthermore, tumor sections were stained with hematoxylin and eosin (H&E) and Ki67 to evaluate the efficacy of different treatments (Figure 7(D)). As expected, H&E and Ki67 staining further confirmed the therapeutic effect of nanoparticles combined with ultrasound and anti-PD-1. The significant tumor regression in Lip-ICG-PFP-cRGD + LIFU + anti-PD-1 group was attributed to the strong immunogenicity of DAMPs released by necrotic cancer cells and recruited tumor infiltrating T cells (CD8^+^). As we know, T cell immunity is known to play a crucial role in tumor immune defense. Importantly, immunofluorescence staining of tumor tissue showed that Lip-ICG-PFP-cRGD combined with LIFU and anti-PD-1 significantly increased the infiltration of cytotoxic CD8^+^ T lymphocytes and IFN-γ release in tumors compared with the other control groups (Figure 7(D)). The proliferation ability of CD8^+^ T cells was also examined through analyzing tumor infiltrating lymphocytes stained with anti-CD3-FITC and anti-CD8-APC antibodies by flow cytometry. As expected, the number of CD8^+^ T cells in the combination group (32%) was higher than that in any other group (Figure 7(E)). These results further demonstrated that LIFU combined with ‘nanobomb’ could effectively stimulate cytotoxic T lymphocytes by immunogenicities tumor-associated antigens and DAMPs, enhancing the anti-tumor response of immune checkpoint blockage therapy. In addition, there were no significant physiological morphology changes in body weight and major organs of mice in each group (Figure 7(B) and S3), which further proved that the nanoparticles had good biocompatibility and low side effects.

## Conclusions

In summary, a novel type of LIFU-activated ‘nanobomb’ was designed, synthesized and surface modified to target tumor cell for sonodynamic therapy and induce immunogenic cell death. we demonstrate that the ‘nanobomb’ can trigger necrosis in cancer cells, which ruptures cell-membrane by acoustic cavitation effect. The nanoparticles were loaded with PFP and ICG to enhance antitumor efficacy and immunological effects. When exposed to low-intensity focused ultrasound, the nanoparticles effectively facilitate the release of damage-associated molecular patterns through burst-mediated cell-membrane decomposition. Therefore, necrosis-inducible ‘nanobomb’ remarkably enhance antitumor immunity by activating maturing dendritic cells. When combined with immune checkpoint blockades, it can significantly improve the inhibition ability to tumor. In addition, the liposome was demonstrated to be a US imaging probe. In summary, the US imaging-guided, LIFU activated ROS production and explosion ‘nanobomb’ can significantly improve anti-cancer efficacy and overcome drug resistance through combination of SDT and immunotherapy, we believe that this is a promising approach for targeted therapy of solid tumor including ovarian cancer.

## Supplementary Material

Supplemental Material

## Data Availability

All data generated or analyzed during this study are included in this published article.
