# Supplementary material for: Acoustic triggered nanobomb for US imaging guided sonodynamic therapy and activating antitumor immunity
Source: Drug Deliv. 2022 Jul 10;29(1):2177–89. doi: 10.1080/10717544.2022.2095058 (PMC9291667; doi:10.1080/10717544.2022.2095058)
Supplement: Supplemental Material [file IDRD_A_2095058_SM3432.docx]

**Acoustic Triggered Nanobomb for US Imaging Guided Sonodynamic Therapy and Activating antitumor immunity**

Mengmeng Li^a#^, Ya Zhu^b#^, Chao Yang^c^, Mi Yang^d^, Haitao Ran^d^, Yefeng Zhu ^d^*, and Wei Zhang^d^*

^a^Department of Obstetrics and Gynecology, the Second Affiliated Hospital of Chongqing Medical University, Chongqing 400010, China;

^b^Department of Obstetrics and Gynecology, Chongqing Traditional Chinese Medicine Hospital of Jiulongpo District, Chongqing 400080, China

^c^Department of Radiology, Chongqing General Hospital, Chongqing, 400013, China.

^d^Department of Ultrasound, the Second Affiliated Hospital of Chongqing Medical University & Chongqing key Laboratory of Ultrasound Molecular Imaging, Chongqing, 400010, China.

*Correspondence: Yefeng Zhu; Wei Zhang

Email: zhuyefeng0575@163.com; [usdoctor@163.com](mailto:usdoctor@163.com)

^#^ Mengmeng Li and Ya Zhu contributed equally to this work.


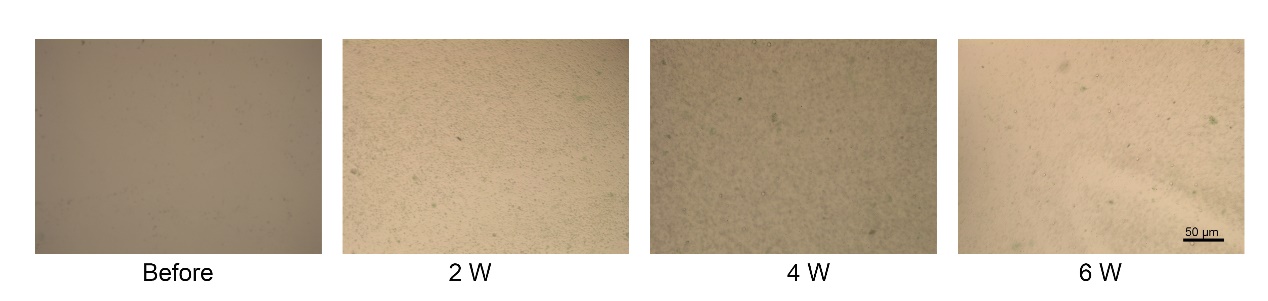


**Figure S1.** Optical images of Lip-ICG-cRGD after LIFU irradiation (2 min, 2-6 W).


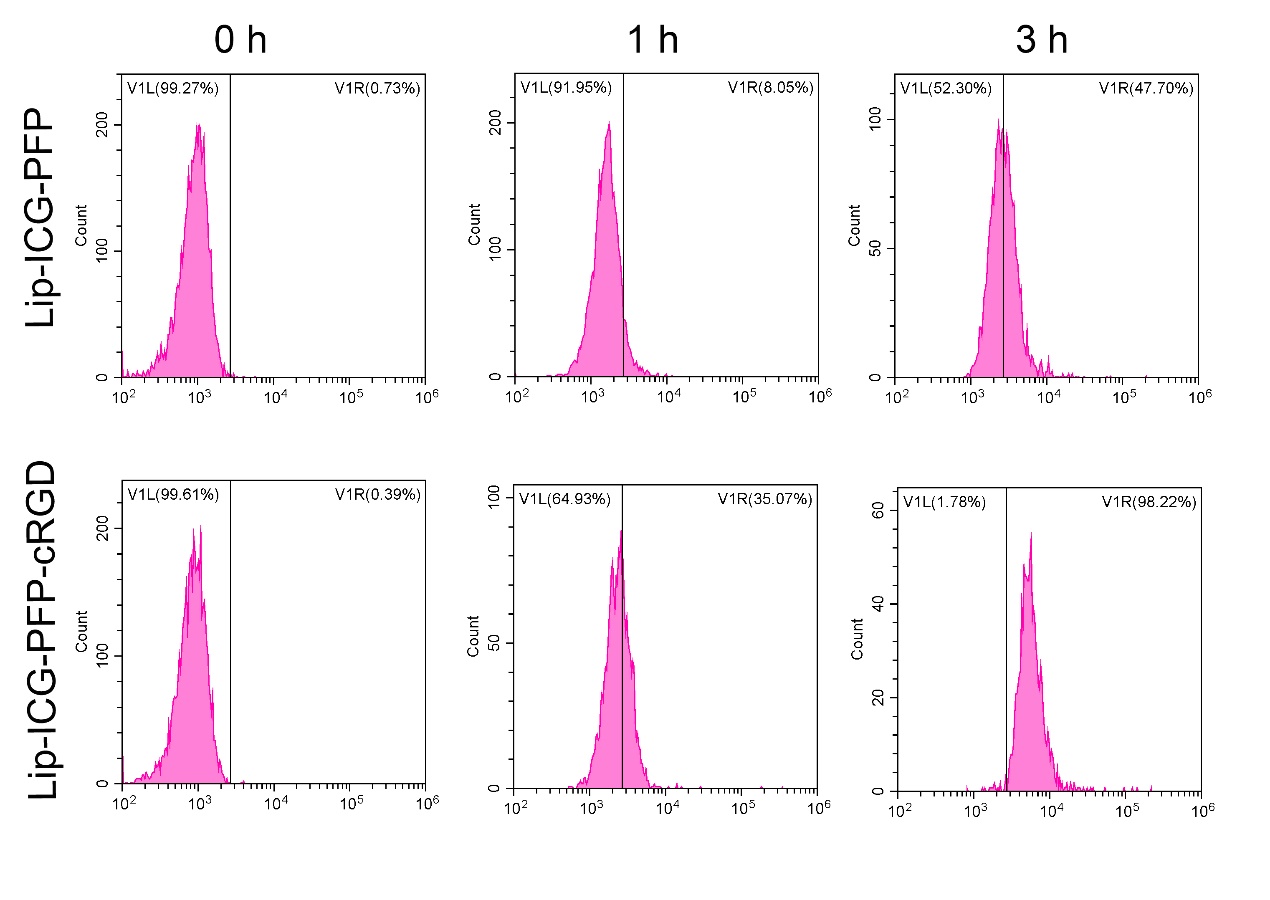


**Figure S2.** Flow cytometry analysis of TC-1 cells after incubation with Lip-ICG-PFP-cRGD and Lip-ICG-PFP nanoparticles for elevated time.


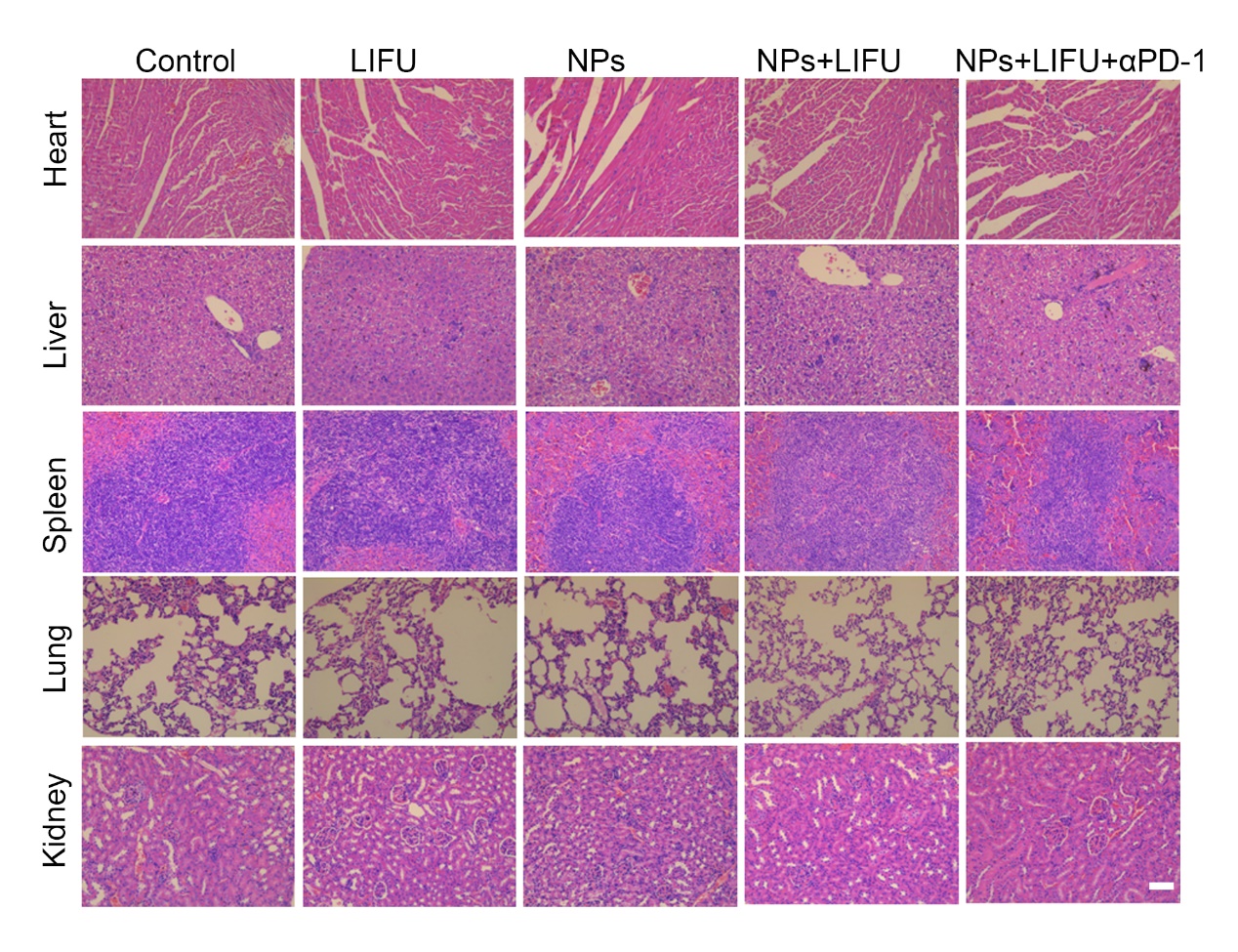


**Figure S3.** The H&E staining of main organs in different treatment groups (scale bar: 50 µm).
